# Insights into Young‐Onset Dementia: A Six‐Year Observational Study from a Regional Tertiary Memory Clinic

**DOI:** 10.1002/alz70857_107611

**Published:** 2025-12-26

**Authors:** Beili Shao, Abigail Rebecca Lee, Elizabeta Mukaetova‐Ladinska, Permesh Singh Dhillon, Kehinde Junaid, Hannah Sargisson, Bruno R Gran, Akram A. Hosseini

**Affiliations:** ^1^ School of Medicine, University of Nottingham, Nottingham, Nottinghamshire, United Kingdom; ^2^ Academic Neurology, Nottingham University Hospitals NHS Trust, Queen's Medical Centre, Nottingham, Nottinghamshire, United Kingdom; ^3^ School of Psychology, University of Leicester, Leicester, Leicestershire, United Kingdom; ^4^ Nottingham University Hospital NHS Trust, Nottingham, Nottinghamshire, United Kingdom; ^5^ Nottinghamshire Healthcare NHS Foundation Trust, Nottingham, United Kingdom; ^6^ Sir Peter Mansfield Imaging Centre, University of Nottingham, Nottingham, Nottinghamshire, United Kingdom

## Abstract

**Background:**

Mild cognitive impairment (MCI) is a transitional state between normal cognitive aging and dementia and presents clinical challenges due to its heterogeneous causes and variable progression. Accurate early diagnosis and subtype classification of MCI are critical for guiding emerging targeted treatments and supporting patients and their families.

**Method:**

This six‐year observational study examined cases of cognitive impairment referred to the regional tertiary memory clinic at Queen's Medical Centre, Nottingham, primarily the Young‐Onset Dementia Clinic. The study, conducted from December 2018 to November 2024, included referrals from Nottinghamshire, Leicestershire, Lincolnshire, and Derbyshire. Data were collected as part of the Cognition and NeuroImaging in Neurodegenerative Disorders (CogNID, IRAS 250525, NCT03861884) study and encompassed demographic, clinical, cognitive, cerebrospinal fluid (CSF) biomarker, and neuroimaging data.

**Result:**

Over six years, 630 individuals were seen at the neurology‐led clinics, and 429 (68.10%) took part in the study. The mean age of participants was 60.08 years, with 71.10% below 65 yr, 54.55% male, and 63.86% Caucasian. The average Addenbrooke's Cognitive Examination (ACE‐III) score was 70.29, with domain sub scores indicating deficits in Attention (13.91/18), Memory (15.05/26), Fluency (7.58/14), Language (21.62/26), and Visuospatial skills (12.45/16). Brain MRI was the most common mode of investigations. Each individual had 1.2 MRI scans and 0.421 FDG‐PET scans on average. CSF analysis was performed in 31.00% of participants. Alzheimer's disease (AD) was the most common diagnosis (*n* = 107, 24.94%). Other significant diagnoses included functional cognitive decline with non‐Alzheimer's pathology (12.59%), frontotemporal dementia (6.99%), COVID‐related cognitive impairment (6.06%), vascular cognitive impairment (4.20%) and dementia with Lewy bodies (2.78%) along with various neurodegenerative, vascular, autoimmune, infectious, metabolic, psychiatric, and movement disorders. 12.82% of participants were considered to have a mixed pathology, while the most common co‐pathology was AD and FTD. Over 6 years of this prospective longitudinal study, mortality rate was 5.13%, with 1.86% of participants transitioning to care homes.

**Conclusion:**

This observational and longitudinal study provides valuable insights into heterogeneous aetiology of MCI including co‐pathologies from real‐world Young‐Onset Dementia Memory Clinics.

